# Using the SEIQR model with epidemic amplifier effect to predict the final outbreak size of the COVID-19 in Dalian, Liaoning province, China

**DOI:** 10.1371/journal.pone.0307239

**Published:** 2024-12-12

**Authors:** Qingyu An, Jun Wu, Wen hui Chen

**Affiliations:** 1 Dalian Center for Disease Control and Prevention, Dalian, Liaoning Province, PR China; 2 School of Public Health, Dalian Medical University, Dalian, Liaoning Province, China; State Key Laboratory for Diagnosis and Treatment of Infectious Diseases, CHINA

## Abstract

**Objectives:**

Early in the outbreak, to predict the final size of the COVID-19 outbreak in Dalian, Liaoning province, China, the finding can be used to provide a scientific reference for timely adjustment of prevention and control strategies.

**Methods:**

Data from COVID-19 patients were collected from August 26 to September 14 2022. Early in the outbreak, a Susceptible-Exposed-Infectious-Quarantine-Recovered (SEIQR) dynamics model with an epidemic amplifier effect, based on the basic model, was developed to fit the data and predict the final size of the COVID-19 outbreak in Dalian, Liaoning province, China. The mean absolute relative error(MARE), root mean squared error(RMSE) and mean absolute error(MAE) were used to assess the predictive capacity of the model.

**Results:**

From 26 August to 14 September 2022, 1132 confirmed cases and infected asymptomatic cases of COVID-19 (558 males and 574 females) were reported in Dalian. There were two epidemic amplifiers in this outbreak, namely, T Market and H Hotel. The outbreak size predicted by the combined application of the SEIQR model with these two amplifiers is 1168.34 cases, and MARE, RMSE and MAE compared to the actual value from September 1 to 14 is 1.894%, 21.473 and 17.492 respectively According to the fitting results of the basic SEIQR model, if there was no epidemic amplifier in this outbreak, the final outbreak size was 349.96 cases, which means that the T Market and H Hotel increased 822 infections through amplification.

**Conclusions:**

Early in the outbreak, it was effective and reliable to use the SEIQR transmission dynamics model with the amplifier effect to predict the final size of the COVID-19 outbreak in Dalian, Liaoning province, China, and the result can provide a theoretical basis for the early closing of the COVID-19 epidemic amplifier sites. Furthermore, the epidemic amplifier effect added to the model can solve the homogeneous mixing hypothesis problem that does not match the actual spread of infectious diseases but commonly used by researchers in the construction process of the dynamic model.

## Introduction

On 5 May 5, 2023, the World Health Organization announced that the coronavirus pandemic is not a global emergency [1]. It does not mean that there are no more COVID-19 cases in countries around the world. COVID-19 remains a global health threat. Because the SARS-COV-2 virus continues to evolve, there is always a risk of outbreaks in some areas.

When COVID-19 outbreak occurred, the biggest concern for policy makers was when the outbreak would reach the peak, and when the outbreak would end, how many people would infected based on the current prevention and control efforts. In this study we focused to solve the last problems.

COVID-19 as an emerging infectious disease, compared to the traditional statistical method, because the dynamic method by reflecting the epidemic rule from the transmission mechanism of the disease can provide a better estimate of the size of the outbreak.

In the study of the dynamics of infectious diseases, the main mathematical model used is the "compartment" model, the basic idea of which was established by Kermack and McKendrick in 1927, called the SIR model. Where S, I, and R represent susceptible, infected, and recovered individuals, respectively [2, 3]. With the deepening of research on the transmission mechanism of the SARS-COV-2 virus, considering that there is an incubation period in COVID-19 transmission, the SEIR model is estimated by adding the E compartment based on the SIR model [4]. With regard to the isolation measures taken in China, domestic researchers add the Q compartment to establish the SEIQR model [5].

In the construction process of the above model, although researchers set different parameters, but they are all based on the same hypothesis, namely the homogeneous mixing hypothesis [6], where the probability of contact is exactly the same for all types of people. This assumption ignores the "amplifiers" in the transmission of infectious diseases [7], which is not only inconsistent with the actual transmission process of the disease, but will certainly greatly affect the eventual size of the epidemic. Therefore, we establish a new susceptible exposed-infected-recovered quarantine model with an "amplifier" effect to predict the size of the outbreak that based on the influence of the prevention and control measures of COVID-19 in 2022, Dalian city, Liaoning province, China. The novelty of the above model could be used by decision makers and researchers in other countries for the COVID-19 outbreak, also could be used for other emerging respiratory infectious diseases that tend to cause outbreak in clusters and lead to further transmission in the community.

## Materials and methods

### Study area

Dalian is the main coastal city in Liaoning province, China, and is a major tourist city located at 38°43′-40°10′N latitude and 120°58′-123°31′E longitude [8]. In 2018, 5.952 million urban residents were registered in Dalian [9].

### Data collection

Daily reporting data for COVID-19 were collected from the China Information System for Disease Control and Prevention. Accessed the system on July 2, 2023 and downloaded the data during August 26 to September 14, 2022 for research purpose. The information contained in the patient’s records was anonymized and identified prior to analysis. The data mainly date of onset were aggregated and analyzed. In China, Covid-19 is a notifiable infectious disease, which is needed when physicians diagnose a case in the course of diagnosis and treatment, and clinicians were required to report COVID-19 cases through the China Information System for Disease Control and Prevention in 2 hours. The cases of COVID -19 included a suspected case, a confirmed case, and an infected case with asymptomatic infection [10].

### Data analysis

#### Basic SEIQR model

In the SEIQR model, individuals were divided into seven compartments, including susceptible individuals(S), isolated susceptible individuals (Sq), latent individuals (E), isolated latent individuals (Eq), positive infected individuals (I), hospitalized positive infected individuals (H) and recovered individuals(R) [4].

According to the mechanism of transmission of COVID-19, for a susceptible individual S, after contacting the latent individual E and/or the positive infected individual I, if the probability of q is tracked as close contacts, and with the probability of 1 − *β* is not infected, then enters the quarantined susceptible individuals Sq, Sq is released after the isolation period of 1 / *λ* and reenters the susceptible individuals S. If the susceptible individual S is not tracked as close contact with the probability of (1-q), but is infected with the probability of *β*, then he or she enters the latent individual E. If the susceptible individual S is tracked with a probability of q and is infected with a probability of *β*, he or she enters the isolated latent individuals Eq.

Latent individual E becomes positive infected I, after the incubation period of 1 / *σ*. For the positive infected individual I, if it is detected with a probability of *θ* through community screening and voluntary medical treatment, then it will enter the hospitalized positive infected individual H. If the positive infected individual I is not detected with probability of (1 − *θ*), he or she will enter the recovered individual R with a recovery rate of r.

The isolated latent individual Eq, after testing positive during the isolation period, will enter the hospitalized positive infected person H. The hospitalized positive infected person H will also enter the recovered individual R with a recovery rate of r.

The description of the system parameters and the corresponding estimation method of parameter value are illustrated in Table 1.

**Table 1:** Description of system parameters and estimation method.

parameters	description	estimation method
*λ*	De-quarantine rate	according to reference literature
*q*	Isolation rate	Retrospective data analysis in Dalian
*β*	transmission probability per contact	curve fitting
*σ*	transition rate of latent individuals to the infected individuals	according to reference literature
*θ*	discovery rate of infected individuals to hospitalized positive infected individuals	Retrospective data analysis in Dalian
*r*_1_, *r*_2_	Recovery rate of the positive infected individual and hospitalized positive infected individuals	according to reference literature
*c*	contact number	Retrospective data analysis in Dalian and curve fitting
*δ*	Rate of isolation to positive test	Retrospective data analysis in Dalian
*d*	Case fatality rate	Retrospective data analysis in Dalian

#### Improved SEIQR model with "amplifier" effect

In order to solve the problem of different probabilities of contact between individuals inside and outside the "amplifier" of the epidemic, we set up two different types of "amplifier" according to their characteristics. The first type is relatively closed, small mobility of population "amplifier", such as schools, factories, etc, the second type is relatively large mobility of population "amplifier", such as shopping malls, supermarkets, etc.

For schools, we assume high-frequency contact between students and teachers during school hours and low-intensity contact with the community for the rest of the day. Saturday and Sunday, because students and teachers are not in school, they do not have close contact with each other and have low intensity contact with the community.

For factories, we assume that working days are Monday to Saturday. During work hours, there is a high frequency of contact between employees and low intensity contact with the community for the rest time of the day. There is a low intensity contact with the community on Sunday.

For shopping centers and supermarkets, we assume that working hours are Monday to Sunday. During work hours, there is a high frequency of contact between employees and also between employees and customers, and a low intensity contact with the community for the rest of the day.

#### Parameters set

For individuals outside the "amplifier", the rate of transfer from one compartment to another is the same as in the basic SEIQR model. The number of contacts in the model is expressed as the community contact number.

For the individual in the "amplifier", the contact number is divided into the effective contact number and the community contact number. Among them, the effective contact number refers to the high frequency contact between individuals in the "amplifier", and the value of that is greater than the community contact number. The duration of effective contact is defined as the time between the infection sources entering the amplifier site and the actual closing of the amplifier site.

**Fig 1:**
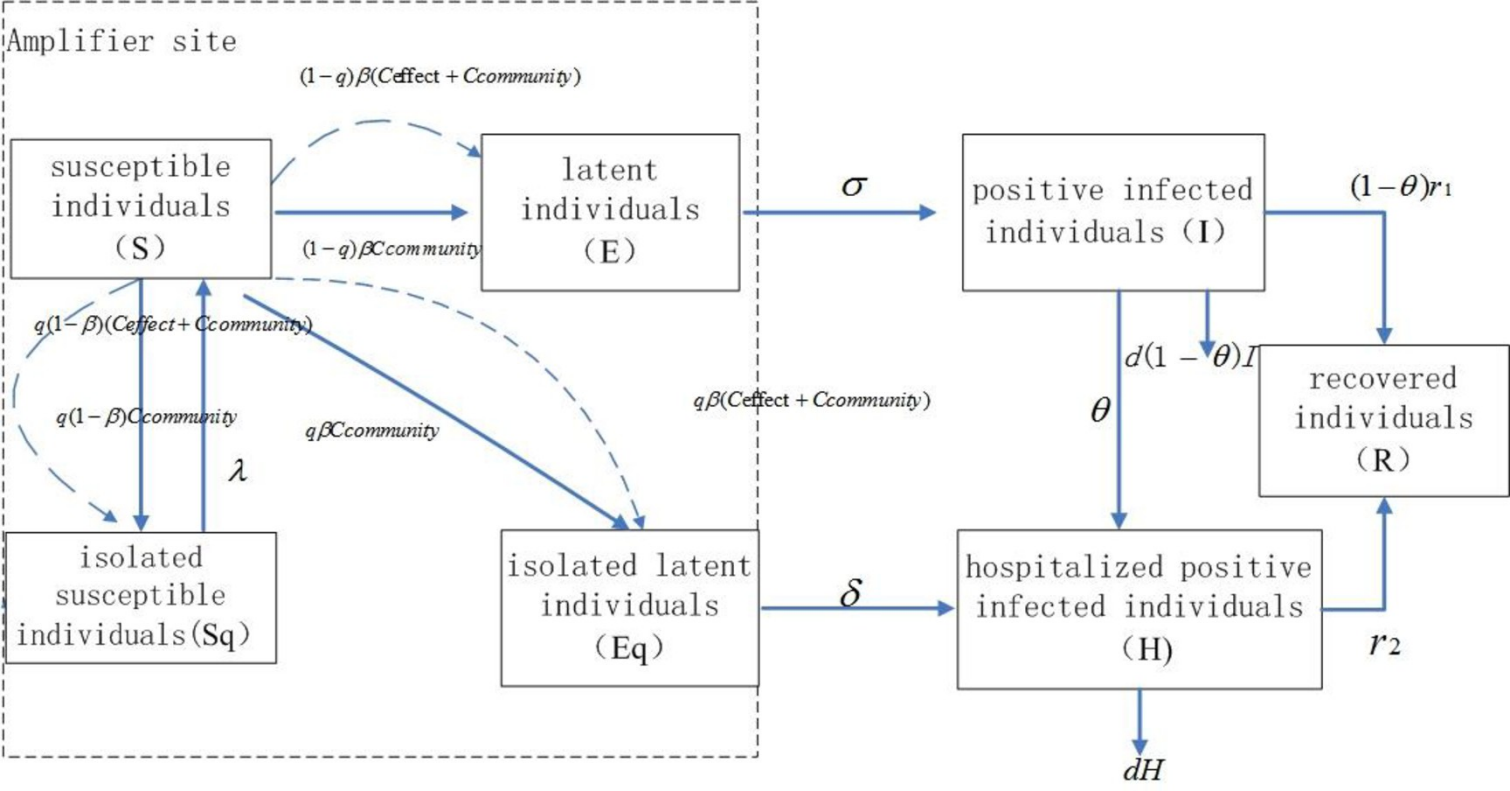
Flow chart of the improved SEIQR model with "amplifier" effect for COVID-19.

The relationship in the improved SEIQR model with the "amplifier" effect between different variables is shown in Fig 1. In Fig 1, the dashed lines show the conversion relationships from one compartment to another within the amplifier site before the amplifier site actually takes closure measure, and the solid lines show the conversion relationship after the amplifier site closes. The equations of the improved SEIQR model with the "amplifier" effect are as follows:

$$\frac{ds}{dt}=-\frac{q(1-\beta )c(t)S(I+E)}{N}-\frac{\left(1-q)\beta c(t)S(I+E\right)}{N}-\frac{q\beta c(t)S(I+E)}{N}+\lambda S_{q}$$

$$\frac{dE}{dt}=\frac{\left(1-q)\beta c(t)S(I+E\right)}{N}-\sigma E$$

$$\frac{dI}{dt}=\sigma E-(1-\theta )r_{1}I-\theta I-d(1-\theta )I$$

$$\frac{dH}{dt}=\delta E_{q}+\theta I-r_{2}H-dH$$

$$\frac{dSq}{dt}=\frac{q(1-\beta )c(t)S(I+E)}{N}-\lambda S_{q}$$

$$\frac{dE_{q}}{dt}=\frac{q\beta c(t)S(I+E)}{N}-\delta E_{q}$$

$$\frac{dd_{H}}{dt}=dH$$

$$\frac{dd_{I}}{dt}=dI$$

where *C*(*t*) has two meanings. Before amplifier site actually take closure, *C*(*t*) means sum of effective contact number during school or work hours and community contact number during the rest of the day. After amplifier site close, *C*(*t*) means the community contact number equals the contact number in the basic SEIQR model. The meaning and estimation method of the other parameters are listed in Table 1.

Berkeley Madonna, v. 9.2.2 was used for parameter fitting and model simulation.

#### Parameter sensitivity analysis

Since the parameters including *λ*, *σ*, r1, r2 are estimated according to reference literature, sensitivity analysis of the parameters is needed to verify the stability and reliability of the model. First of all, each parameter range was evenly divided to get 1000 parameter values. The value of one parameter was changed each time and the other parameter values are maintained to their nominal value, then the cumulative number of cases is observed [11]. Since the above four parameters were set identically in the SEIQR model with the T market and the H hotel amplifier, in the parameter sensitivity analysis section, the cumulative number of cases is observed until August 31 2022. Secondly, a curve with parameter position as the horizontal coordinate and cumulative incidence number of cases as the vertical coordinate is drawn. Third, if a parameter with a large slope, the cumulative number of cases when the parameter value (the above divided into 1000 equal parts) is $\bar{\chi }$, $\bar{\chi }+s$, $\bar{\chi }-s$ is calculated respectively [12], then calculated mean absolute relative error(MARE), root mean squared error(RMSE) and mean absolute error(MAE) to compared with the cumulative number of cases by the actual value.

#### Evaluation index of predictive capacity of the model

The criterion for comparing the predictive capacity of the model is the mean absolute relative error(MARE), root mean squared error(RMSE), mean absolute error(MAE), given by the respective expressions:

$$e=\frac{1}{n}\sum_{t=1}^{n}\left|\frac{x_{t}-{\hat{x}}_{t}}{x_{t}}\right|\times 100\%$$

$$RMSE=\sqrt{\frac{1}{n}\sum_{t=1}^{n}(x_{t}-{\hat{x}}_{t}}{)}^{2}$$

$$MAE=\frac{1}{n}\sum_{t=1}^{n}\left|{\hat{x}}_{t}-x_{t}\right|$$

where *x*_*t*_ and ${\hat{x}}_{t}$ denote the observed and fitted values for that point in time, *n* denotes the total number of time series. Microsoft Excel statistical package was used for data analysis.

### Ethics statement

This study was approved by the ethics committee of Dalian center for control and prevention. Dalian COVID-19 data during August 26 to September 14 2022 were obtained from the China information system for disease control and prevention. The need for consent was waived by the ethics committee, because there were no ethical issues relevant to the study design and no individual-level analysis was performed. The information contained in the patient’s records was anonymized and identified prior to analysis. The data mainly date of onset were aggregated and analyzed.

## Results

### 1. Descriptive analysis

An outbreak of COVID-19 that lasted from August 26 to September14, 2022, occurred in Dalian, Liaoning Province, China. During the above period, 1,132 confirmed cases and asymptomatic infected cases of COVID-19 (558 males and 574 females) were reported. The cases were distributed in eight of the 12 districts (Table 2). The median age of the patients was 48 years (range: 5 months to 93 years).

**Table 2:** Shows the number of cases reported in the Dalian district during a COVID-19 outbreak (26 August to 14 September 2022).

district	Cumulative number of cases	Cumulative incidence rate(/100,000)
Zhong shan district	448	115.639
Xi gang district	282	92.693
Gan jing zi district	222	14.449
Sha he kou district	85	12.717
Gao xin yuan district	40	12.960
Jin pu xin district	29	1.873
Pu lan dian district	24	3.830
Lv shun kou district	2	0.564
overall	1132	15.247

### 2. Estimation of parameters of the improved SEIQR model with "amplifier" effect

On 29 August2022, we combined with the results of the epidemiological investigation and the development trend of the outbreak, finding the T market to be the amplifier. The T Market is the largest wholesale and retail market for vegetables, fruits and seafood in the Zhongshan district, with a daily customer of 20,000 people. So, the improved SEIQR model with "amplifier" effect was created on August 30th, 2022, the parameter adjustment was completed until 24 o’clock on August 31st, 2022.

According to retrospective data analysis of epidemiological investigation, the first reported case on the T market was on 26 August. The market had a great price reduction promotion on August 27 and 28 and was scheduled to close at 24 p.m. on August 28, but it was not completely closed, from August 29 to August 30, there were still some vendors selling goods, the number of customers was significantly lower than usual. Therefore, the situation above the COVID-19 outbreak in Dalian is divided into four periods. August 26, August 27 to 28, August 29–30, August 31 to the end. The effect time of the epidemic amplifier was set from August 26 to August 30.

On 20 to 22 August, the SARS-COV-2 nucleic acid testing was carried out in Dalian on a regular day. When the mixed tube appeared positive, new cases were found through close contact screening. Therefore, the number of daily reported cases in the early stage of the outbreak did not exist in all cases, but those discovered on that day, and there could be cases that have not yet been tested. In the epidemic process, all infected persons were identified through community screening, close contact screening, key population screening, and high-risk area screening. Thus, we applied the first three periods, namely August 26, August 27 to 28, August 29–30, to fit the model and evaluated the model by comparing the fitting values and predicted values on the 5th to 6th day after the outbreak began. If the fitting effect was poor, the coefficients were adjusted from the first part to the third part until a satisfactory fitting effect was achieved. Finally, the size of the outbreak was predicted by the finalized model.

Simultaneously considering the limited range of activities and contacts of a person infected with COVID-19 during the period of infection, and the probability of going to the market every day, it is unrealistic to set 100,000 (20,000*5) as the initial susceptible individual, so in this study we estimate that the initial S value would be 20,000. According to the results of the analysis of previous outbreak data in our city, one person can be in effective contact with 50 people per day, so *C*_community_ equal to 50. The effect value on August 26 sets 50 to 200, and the effect value on August 27 to 28 sets at a value twice on August 26, equal to from 100 to 400. From 24 o’clock on 29 August all buses and subways in the main urban Dalian city have been suspended, advocating working at home, but it was still possible to drive private cars to go to work. Affected by the implementation of the above prevention and control measures, the number of contacts with effect on 30 August decreased compared to the previous period. In this study, we set the value of *the C*_*effect*_ from 29 to 30 August at half the value on 26 August, equal to 25 to 100. From 31 August 2022, with cooperation in the implementation of the above prevention and control measures has been further strengthened, the number of community contacts of individuals who chose to work at home decreased significantly. In this study, the *C*_community_ after 31 August 2022 is established as 10.

Based on epidemiological findings, we set the initial I on the T market at 1. Meanwhile, according to the reference, the latent individuals is 10 times that of the infected person, so we estimate the initial E is 10. The initial Sq sets at 282 based on actual control measures taken as of 24 hours on August 26. The initial R, H, and Eq are all set as 0. N is the sum of initial susceptible individuals(S), isolated susceptible individuals (Sq), latent individuals (E), isolated latent individuals (Eq), positive infected individuals (I), hospitalized positive infected individuals (H) and recovered individuals(R), equal to 20293. In this study, we set the integer N to 21000.

If there was no positive test in isolated individuals during 10 days of medical observation [13], the isolation was lifted, so *λ* equals 1/10.

Based on the results of whole genome sequencing (second generation sequencing), the evolutionary branch BA.5.2.1 caused the COVID-19 outbreak in Dalian during August 26 to September 14. Because the average incubation period of COVID-19 caused the Omicron is 3.03 to 4.2days [14], so *σ* equal to1/5.

According to the results of the analysis of previous outbreak data in our city, the isolation rate of susceptible individuals, latent individuals, and positive infected individuals is very high, up to 99.9%. So q and *θ* equal to 0.999. Meanwhile, the shortest period of medical observation from isolation to positive test is the same day, that is, 0 days, the longest is 12 days, and most of them are 0–2 days. So in this study, we set *δ* as 1/12.

According to the retrospective analysis of the clinical data from the COVID-19 cases from January 1, 2021 to December 31, 2021 in Dalian, the median date of onset to discharge in asymptomatic and mild cases was 16 days, and the duration of the disease was 6 to 54 days in all cases, therefore, r1 equals 0.07 (1/16), r2 equal to 0.019 (1/54).

On 25 August 2022, there have been no deaths caused by COVID-19 in Dalian, d is set at a very low value, equal to 0.000001.

According to the results of the analysis of the previous outbreak caused by VOC/Delta in our city, the parameter *β* was 0.03. Since the transmissibility of the Omicron variant is greater than that of the VOC/Delta, it will be greater than 0.03. Therefore, in this study we set the initial *β* as 0.03, then increase by 0.02 each time until the curve fits good. On this basis, we increased by 0.001 each time until the curve fit properly.

Table 3 shows the cumulative number of COVID-19 cases fitted on 31 August 2022 under different *β* and *C*_*effect*_. Compared with the actual value in 228 cases, when *β* value equals 0.13, *C*_*effect*_ value is equal to 0.13, the *C*_*effect*_ value is equal to 150-300-75, the absolute relative error, absolute error is the smallest respectively(5.77%, 13.15), the second small one is when β value equals 0.11, and the *C*_*effect*_ value is 200-400-100, the third small one is when β value equals 0.11, the *C*_*effect*_ value equals 0.11, the *C*_*effect*_ value equals 150-300-75. It can be seen in Table 2 that when the effect is fixed, the fitting value increases with increasing *C*_*effect*_; similarly, when the increase of *β*; similarly, when *β* is fixed, the fitting value increases with increasing effect. Therefore, we selected the fitting value less than 228 cases in the above three groups, namely the group with *β* value equal to 0.11, *C*_*effect*_ value equal to 0.11, the effect value equal to 0.11, the effect value equal to 150-300-75, and explored the fitting value closer to the actual value by fixing the *β* value, namely equal to 0.11, and increasing the effect value, increased by 10 each time. Then we again selected the effect value less than 228 cases, namely when the effect value is equal to 170-340-85 (see Table 4) and explored the fitting value more closer to the actual value by fixing the effect value and increasing the *β* value, increased by 0.001 each time. It can be seen in Table 5 that when *β* value equal to 0.113, the cumulative number of fit cases as of August 31 was 227.82, Very close to the actual value of 228 cases.

**Table 3:** The combined fitted number of cases of COVID-19 as of 31 August 2022 under different *β* and *C*_*effect*_ values.

*β* value	*C*_*effect*_ value			*C*_community_ value	fitting cumulative number of cases as August 31	absolute relative error(%)	absolute error
	26-Aug	August 27 to 28	August 29 to 30				
0.03	50	100	25	50	33.08	85.49	194.92
	100	200	50	50	46.75	79.5	181.25
	150	300	75	50	59.27	74	168.73
	200	400	100	50	70.67	69	157.33
0.05	50	100	25	50	50.85	77.7	177.15
	100	200	50	50	73.79	67.64	154.21
	150	300	75	50	94.76	58.44	133.24
	200	400	100	50	123.89	45.66	104.11
0.07	50	100	25	50	68.66	69.89	159.34
	100	200	50	50	101	55.7	127
	150	300	75	50	130.75	42.65	97.25
	200	400	100	50	168.8	25.96	59.2
0.09	50	100	25	50	86.65	62	141.35
	100	200	50	50	126.58	44.48	101.42
	150	300	75	50	167.08	26.72	60.92
	200	400	100	50	202.47	11.2	25.53
0.11	50	100	25	50	104.76	54.05	123.24
	100	200	50	50	156.35	31.43	71.65
	150	300	75	50	203.65	10.68	24.35
	200	400	100	50	247.6	8.6	19.6
0.13	50	100	25	50	122.95	46.07	105.05
	100	200	50	50	184.41	19.12	43.59
	150	300	75	50	241.15	5.77	13.15
	200	400	100	50	293.23	28.61	65.23

**Table 4:** The cumulative fitted number of cases of COVID-19 as of August 31, 2022 under different *C*_*effect*_ value with *β* equal to 0.11.

*C*_*effect*_ value			*C*_community_ value	fitting cumulative number of cases as August 31	absolute relative error(%)	absolute error
26-Aug	August 27 to 28	August 29 to 30				
160	320	80	50	212.94	6.61	15.06
170	340	85	50	221.83	2.71	6.17
180	360	90	50	230.56	1.12	2.56

**Table 5:** The combined fitted number of cases of COVID-19 as of 31 August 2022 under different *β* value with *C*_*effect*_ equal to 170-340-85.

*β* value	fitting cumulative number of cases as August 31	absolute relative error(%)	absolute error
0.111	223.83	1.83	4.17
0.112	225.82	0.96	2.18
0.113	227.82	0.08	0.18
0.114	229.95	0.86	1.95

On 4 September 2022, we combined with the results of the epidemiological investigation and the development trend of the outbreak, the H hotel was also found to be an amplifier. The Hotel H is located across the street from the Dalian International Convention Center and has 365 guest rooms and suites; the grand ballroom of 1688 square meters and six multifunction halls. Based on the findings of the epidemiological investigation, the initial value of the susceptible individual S was 15,600, the initial I in the hotel H was 5 and the initial E was 50. The method of estimating the initialization of E is the same as the "T market amplifier". The initial Sq is set at 324 based on actual control measures taken at 24 o’clock on September 4 2022. The initial R, H, Eq are set at 0. N is an integer of 16000. Since the outbreak with the hotel H amplifier was caused by the same Omicron variant as the T Market amplifier, we set the initial *β* as 0.113. According to the parameter of the above model in the T market amplifier, the community is set as 10. The mobility of the population in the hotel is relatively smaller than that of the market (170-340-85), but mainly higher than the contact number of the community (10). Therefore, in this study we set the effective contact number from 10 to 85. The last exposure to the source of infection in the first case was on 30 August, and the hotel adopted closed management at 24:00 on 2 September 2022, so the amplifier effect of this hotel lasted for 3 days.

The other parameters set as the same as the model in the above. Parameter estimation process as the same as the model with the T market amplifier.

Compared to the difference between the actual values from 2 September (453.05) and the fitting values of the SEIQR model with the gain of the T market amplifier (340.55), that is, 113.45, when the *C*_*effect*_ value equals 50, the absolute relative error and absolute error is the smallest respectively (0.84%,0.95) and the fitting value(112.50) very close to the actual value(see Table 6).

**Table 6:** The combined fitted number of cases of COVID-19 as of September 2, 2022 with different *C*_*effect*_ value with *β* equal to 0.113.

*C*_effect_ value	*C*_community_ value	Fitting cumulative number of cases as September 2	absolute relative error(%)	absolute error
10	10	57.24	49.55	56.21
15	10	65.53	42.24	47.92
20	10	73.59	35.13	39.86
25	10	81.42	28.23	32.03
30	10	89.03	21.52	24.42
35	10	96.43	15	17.02
40	10	103.61	8.67	9.84
45	10	110.6	2.51	2.85
50	10	112.5	0.84	0.95
55	10	118.91	4.81	5.46

**Fig 2:**
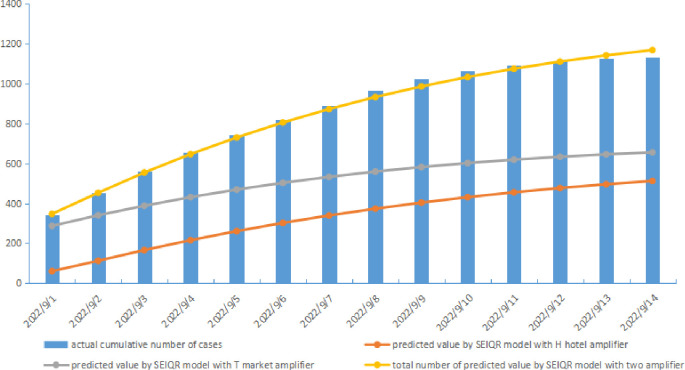
The daily predicted cumulative number of COVID-19 cases using the SEIQR model with the T market and the H hotel amplifier.

Based on the parameters finally determined above, the outbreak size predicted by the combined application of the SEIQR model with the T market amplifier and the H hotel amplifier is 1168.34 cases, and MARE, RMSE and MAE compared to the actual value from September 1 to 14 is 1.894%, 21.473 and 17.492 respectively (see Fig 2 and Table 7).

**Table 7:** Evaluation result of predictive capacity of the SEIQR model with T market and H hotel amplifier.

	actual cumulative number of cases	predicted value by SEIQR model with H hotel amplifier	predicted value by SEIQR model with T market amplifier	total number of predicted value by SEIQR model with two amplifier
2022/9/1	342	60.330	287.167	347.497
2022/9/2	454	112.500	340.554	453.054
2022/9/3	560	166.292	388.428	554.720
2022/9/4	655	215.766	431.221	646.987
2022/9/5	742	261.036	469.336	730.373
2022/9/6	820	302.284	503.151	805.434
2022/9/7	893	339.713	533.014	872.727
2022/9/8	969	373.537	559.250	932.788
2022/9/9	1025	403.971	582.161	986.132
2022/9/10	1063	431.226	602.023	1033.249
2022/9/11	1091	455.509	619.094	1074.603
2022/9/12	1116	477.020	633.612	1110.632
2022/9/13	1126	495.951	645.798	1141.749
2022/9/14	1132	512.487	655.854	1168.341
MARE(%)	1.894			
RMSE	21.473			
MAE	17.492			

### 3. Parameter sensitivity analysis

**Fig 3:**
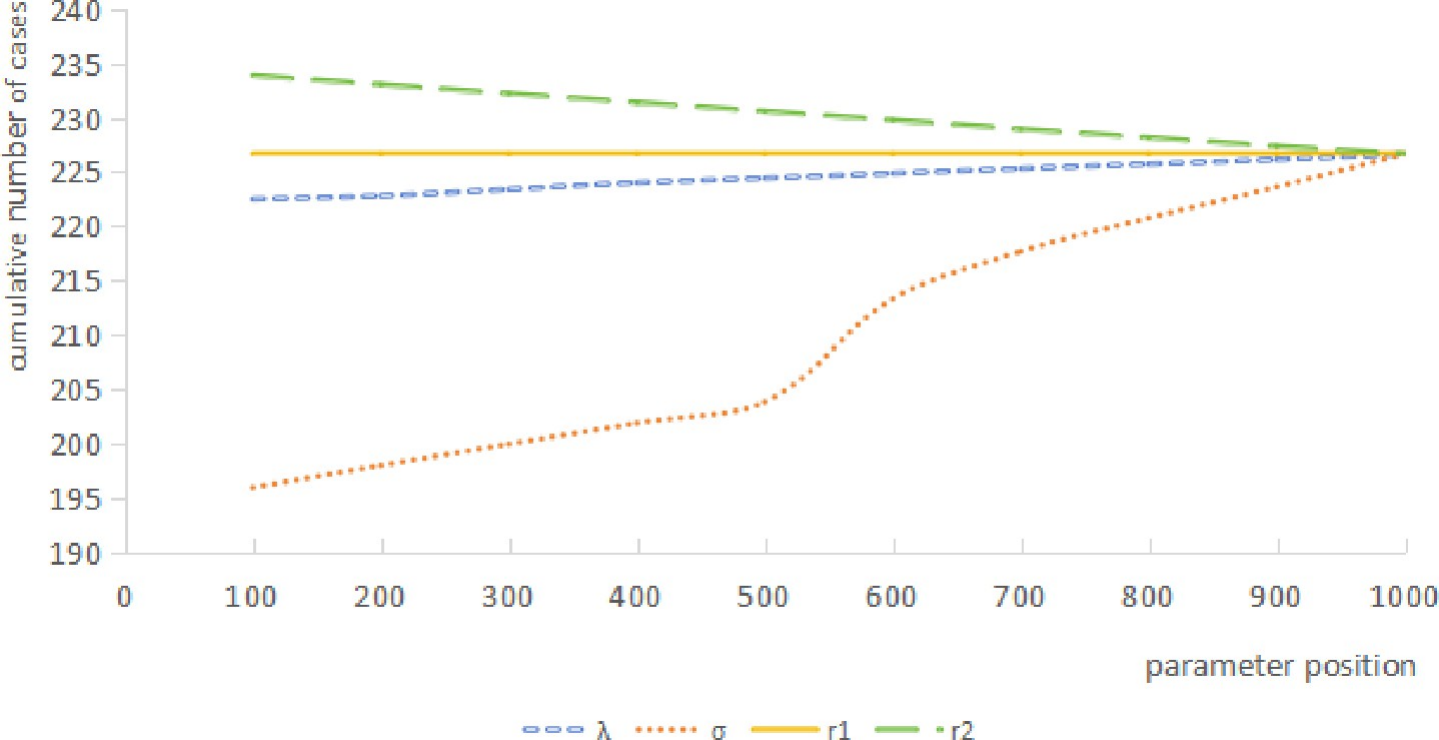
*λ*, *σ*, r1, r2 sensitivity analysis results of the SEIQR model.

Among the four parameters of sensitivity analysis, it can be seen that the sensitivity of parameter r1 is low, and the curve is horizontal and straight, that is, the cumulative number of cases does not change with the change of parameter position. The other three parameters have certain sensitivity, among which *λ* and *σ* show an increasing trend of cumulative number of cases, while r2 shows a decreasing trend of cumulative number of cases with an increasing rank (see Fig 3).

**Fig 4:**
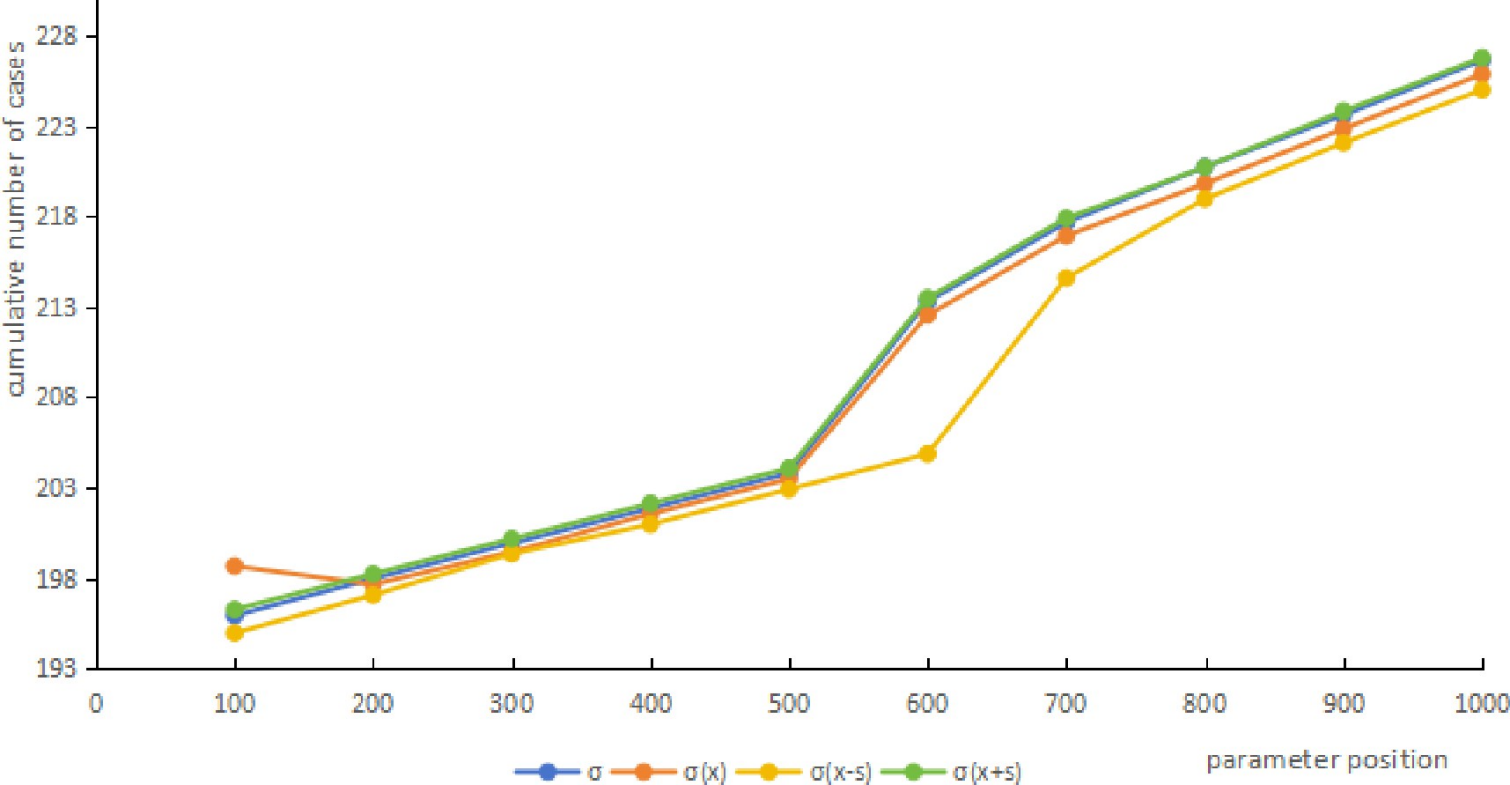
*σ* sensitivity analysis results of the SEIQR model.

Because the slope of the *σ* is larger than other parameters, the cumulative number of cases when the parameter value is $\bar{\chi }$, $\bar{\chi }+s$, $\bar{\chi }-s$ is calculated respectively, then compared with the number of cases calculated by the actual value. The results showed that the cumulative number of cases all within $\bar{\chi }\pm s$, indicating that the *σ* values were reasonable in this study(see Fig 4 and Table 8).

**Table 8:** *σ* sensitivity analysis results of the SEIQR model.

parameter position	cumulative number of cases			
	*σ*	*σ*(x)	*σ*(x-s)	*σ*(x+s)
100	195.91	198.65	194.95	196.25
200	197.96	197.63	197.05	198.22
300	199.92	199.44	199.33	200.17
400	201.86	201.55	200.97	202.13
500	203.80	203.48	202.91	204.07
600	213.31	212.56	204.86	213.5
700	217.68	216.94	214.61	217.92
800	220.76	219.84	218.99	220.76
900	223.63	222.87	222.09	223.85
1000	226.67	225.91	225.04	226.81
	MARE(%)	0.390	0.970	0.106
	RMSE	1.056	3.044	0.235
	MAE	0.811	2.07	0.218

### 4. Evaluation of the "amplifier" effect

In this study, we evaluated the effect of amplifiers in this COVID-19 outbreak in Dalian by building an SEIQR model and comparing its result with the above results of the SEIQR model with the effect of the amplifier. In the basic SEIQR model, except that the contact number only includes the community contact number(50), other parameters and initial value settings are the same as the SEIQR model with the T market amplifier on August 26, 2022. On 31 August 2022, the initial value of the SEIQR model was added with the H model, and the number of community contacts was adjusted to 10.

**Fig 5:**
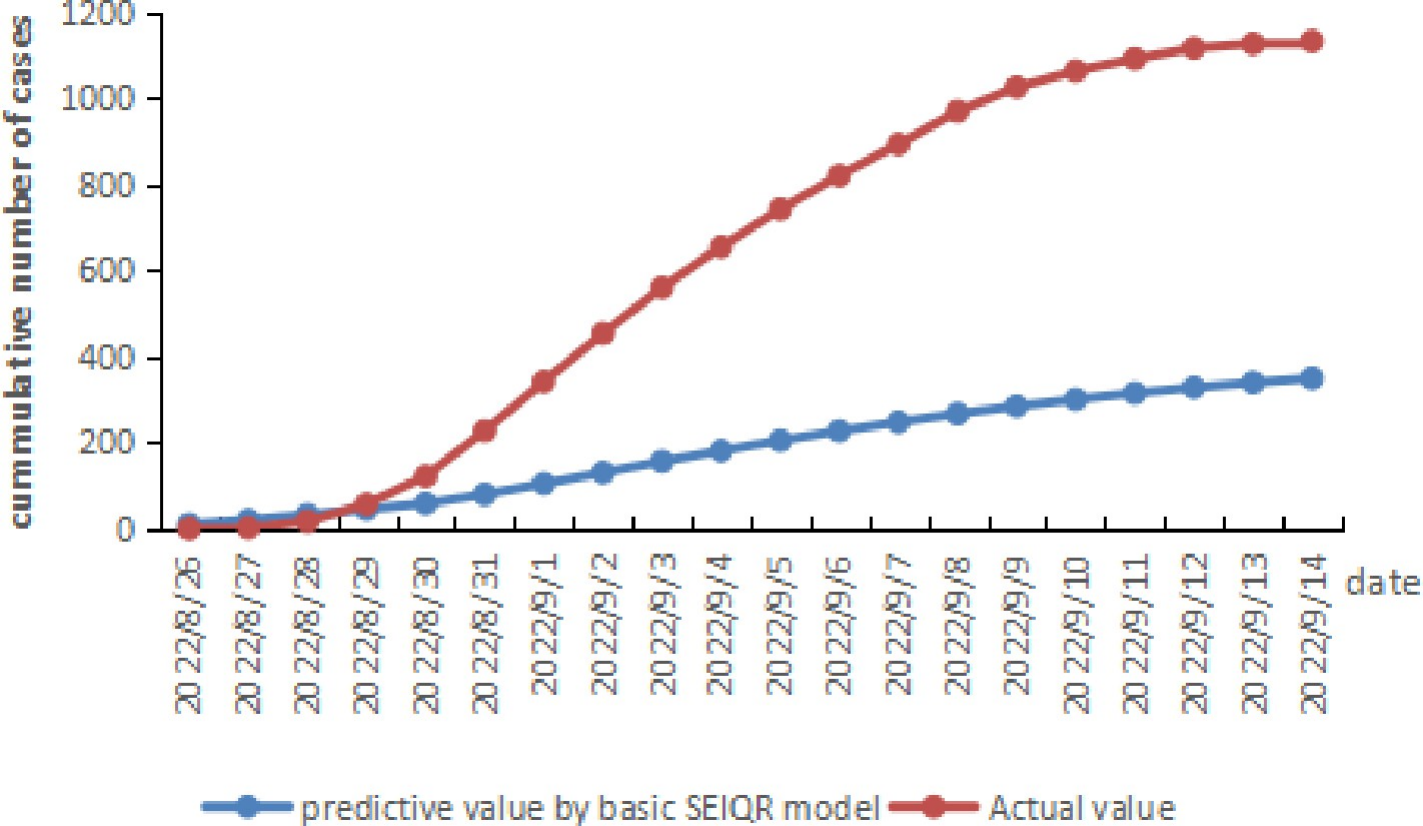
The daily predicted cumulative number of cases of COVID-19 using the basic SEIQR model.

According to the predicted results of the basic SEIQR model, if the total number of people related to the T market (21,000 people) and that related to the H Hotel (16,000 people), that is, 37000 people, spread from person to person through community contact without the amplifier, 349.96 individuals would eventually be infected, the infection rate is 0.95%. In the outbreak, the T market and the H Hotel increased the number of infections by 822 by amplification (see Fig 5).

## Discussion

An outbreak of new coronavirus diseases (COVID-19) in 2019 in Wuhan, China, has spread rapidly throughout the country. Although the World Health Organization declared on 5 May that the COVID-19 pandemic is no longer a global emergency, worldwide, almost 2.3 million new cases and nearly 15,000 deaths were reported during April 24 to 21, 2023, and 161 countries reported at least one case [15].

Since the first imported case of COVID-19 reported on 20 January 2020, on 25 August 2020, a total of 1798 confirmed and asymptomatic infected cases of COVID-19 were reported in Dalian, including 5,515 imported cases from overseas. The number of cases of COVID -19 reported is less than that of Shanghai Pudong New Area [16], Wuhan [17], Jilin Changchun [18] in China during roughly the same period.

In this study, we found that T Market and H Hotel are two amplifiers in the COVID-19 outbreaks that occurred in Dalian, Liaoning Province, China, and lasted from 26 August to 14 September 2022. The amplifier of infectious disease transmission process means that when there is an infectious source in a gathering place where the population is crowed, the disease is transmitted to others through high-frequency contact between persons in the gathering places, and infectious individuals then cause community transmission through affecting family members. For example, school is a special crowded place, students and teachers in the closed space of the classroom for a long time and face-to-face contact, this frequent close contact increases the risk of transmission of diseases. Therefore, if there is a source of infection in the school, the probability of students and teachers contacting infected people in the school is much higher than outside the school. For another example, shopping mall where the population was crowed and floating over, if it is in winter and ventilation is not good, the probability of contact with infected individuals in the shopping mall is much higher than outside the mall. Infected individuals in schools and shopping malls spread to the community by affecting family members.

T Market and H Hotel increased the number of infections to 822 cases, representing 72.61% of the total size of the outbreak. Therefore, to reduce the incidence rate, the amplifier site should be closed as early as possible to shorten the effect time of the amplifier site.

In this study, we evaluated the COVID-19 transmission capacity in Dalian City from August 26 to September 14,2022 by developing a SEIQR transmission dynamics model with amplifier effect in initial phase of the outbreak. The predicted outbreak size is very close to the actual value. The mean absolute relative error is only 1.894%. The results show that the SEIQR transmission dynamics model with the amplifier effect is suitable to evaluate the transmission capacity of emerging respiratory infectious diseases with an easy transmission route such as COVID-19. The reasons are as follows. First, for emerging infectious diseases, the knowledge about the disease is not fully understood, so compared to the traditional statistical method, the dynamic method can better reflect the epidemic law of the disease from the point of view of the transmission mechanism. Second, for emerging respiratory infectious diseases, due to the population being susceptible and having an easy transmission route, it is easy to spread in gathering places such as schools and shopping malls. So, the dynamic model with the function of "amplifier" can make the theoretical estimation of the mathematical model of the epidemic situation of emerging respiratory infectious diseases more consistent with the actual incidence situation. In this study, the MARE of the SEIQR transmission dynamics model with amplifier effect less than Adaptive Neuro-Fuzzy Inference System (ANFIS) model [19]. Abdallah Alsayed et al [19] used ANFIS model to provide short-time forecasting of the number of infected cases in Malaysia 2020. According to this study, the mean absolute percentage error namely MARE is 2.45%.

In this study, the root mean squared error and mean absolute error of the SEIQR transmission dynamics model with the amplifier effect is 21.473 and 17.492 respectively, higher than that of an exponential decay model applied to the cumulated and weighted average daily growth rate [20], ARIMA model [21], and less than cellular automata model [22].

Nicola Bartolomeo et al [20] used an exponential decay model applied to the cumulated and weighted average daily growth rate(WR) to estimate the number of cases and peak of the COVID-19 outbreak in Italy. In the process of establishing the above exponential decay model, the authors calibrated the parameters of the exponential decay model to the reported data in Hubei Province of China between January 21,2020 and March 18,2020. According to this study, the RMSE for exponential decay model with WR model in four-time intervals from February 27 to April 07, 2020, is 0.019,0.018,0.016 and 0.014 respectively.

Sherry Mangla et al [21] used an ARIMA model to short-term forecast cumulative number of infected cases in COVID-19 outbreak 21 December 2020 to 4 January 2021 in India and its five most affected states. According to this study, the RMSE for ARIMA model is 0.2.

Wu Zeyang et al [22] used an cellular automata model to simulate the small-scale outbreak of COVID-19 in Heilongjiang province of china. The MAE forcellular automata model is 30.37.

For emerging respiratory infectious diseases, due to the general susceptibility of the population, can spread rapidly, there is a risk of global pandemic [23], and will continue to occur in the future [24]. Emerging infectious diseases are estimated to account for more than 12% of all infectious diseases [25]. In emerging infectious diseases, emerging respiratory infectious diseases accounted for 35%. Due to the prevalence of emerging respiratory infectious diseases, such as the COVID-19 pandemic, it can have a negative impact on economic and social development around the world; therefore, in the case that the knowledge about the disease is not fully understood, the construction of a transmission dynamic model that can reflect the actual incidence situation, it has a significant public health importance to understand the transmission mechanism and the transmissibility of an emerging virus, considering that preventive and control measures should be taken in time to reduce the scale of the outbreak and reduce the risk to public health and socioeconomic development. Of course, it is challenging to construct a more realistic transmission dynamic model. In the case of COVID-19, with the continuous variant of the SARS-COV-2, if the transmission speed of the virus has significantly changed compared with that of the past variant, the dynamics of transmission model which the parameters are estimated based on previous variant no longer applicable, the short-term prediction like exponential decay model applied to the weighted and averaged growth rates [20] can be alternative used.

## Conclusions

During the outbreak, the use of the SEIQR transmission dynamics model with the amplifier effect to predict the final size of the COVID-19 outbreak in Dalian, Liaoning province, China was effective and reliable, and the result can provide a theoretical basis for early closure of the COVID-19 epidemic amplifier sites of the COVID-19 epidemic and the novel of the above model with the amplifier effect can be used to construct the transmission dynamics model of other emerging respiratory infectious diseases. Furthermore, the epidemic amplifier effect added to the model can solve the homogeneous mixing hypothesis problem that does not match the actual spread of infectious diseases but commonly used by researchers in the construction process of the dynamic model.

## Supporting information

S1 Data Daily onset cases of COVID-19 in Dalian, Liaoning province, China, between 26 August 2022 and 14 September August 2022. (XLSX)

## References

[1] Statement on the fifteenth meeting of the IHR (2005) Emergency Committee on the COVID-19 pandemic.https://www.who.int/news/item/05-05-2023-statement-on-the-fifteenth-meeting-of-the-international-health-regulations-(2005)-emergency-committee-regarding-the-coronavirus-disease-(covid-19)-pandemic.

[2] Ming-jin Xue, Zhao-wei Huang, Yu-di Hu, Jin-lin Du, Zhi-gang Huang. Research progress of infectious disease dynamics models. Prev Med,2022,34(1):53–57.

[3] Yu Yue, Shi Man-man, Hu Man-feng, Zhang Jing-xiang. Assessing the Impact of Continuous Vaccination and Voluntary Isolation on the Dynamics of COVID-19: A Mathematical Optimal Control of SEIR Epidemic Model. Computational Intelligence and Neuroscience Volume 2022, Article ID 3309420,1–13. doi: 10.1155/2022/3309420 35665296 PMC9161137

[4] Yun-jun Zhang, Yuan Zhang, Chong You, Xiao-hua Zhou. Review on the study of spreading of the COVID-19 based on dynamic models. Chin J Med Sci Res Manage, 2020, 33 (Z1): 36–40. doi: 10.3760/cma.j.cn113565-20200214-00007

[5] Sheng-li Cao, Pei-hua Feng, Peng-peng Shi. Study on the epidemic development of COVID-19 in Hubei province by a modified SEIR model. Journal of Zhejiang university (medical sciences), 2020, 178–184. doi: 10.3785/j.issn.1008-9292.2020.02.05 32391661 PMC8800716

[6] He Shao-bo. Peng Yue-xi. Sun Ke-hui. SEIR modeling of the COVID-19 and its dynamics. Nonlinear Dyn (2020) 101:1667–1680. doi: 10.1007/s11071-020-05743-y 32836803 PMC7301771

[7] Hai-feng Wang, Ya-fei Li, Ying-ying Wang, Ming-yang Yu, Wei Fan, Yi-fei Nie, et al. Characteristics of 365 infected cases for COVID-19 outbreak caused by SARS-CoV-2 Delta variant in 2022. Henan J Prev Med, 2022, 33(8):561–564.

[8] Qing-yu An, Wei Yao, Jun Wu. Predicting clinically diagnosed dysentery incidence obtained from monthly case reporting based on meteorological variables in Dalian, Liaoning province,China,2005–2011 using a developed model. Southeast Asian J Trop Med Public Health. 2015;46:285–95. 26513932

[9] Baidu. Dalian. https://baike.baidu.com/item/%E5%A4%A7%E8%BF%9E/152852?fr=aladdin. Accessed 28 Aug 2020.

[10] National health commission of the people’s republic of china. (2020) COVID-19 Prevention and Control Plan (Sixth Edition). http://www.nhc.gov.cn/xcs/zhengcwj/202003/4856d5b0458141fa9f376853224d41d7.shtml. Accessed 23 Aug 2020. 10.46234/ccdcw2020.082 PMC8392946 34594648

[11] Yue Zhang, Ruo-meng Bi, Jian-xin Ma, Qian Li, Zheng Zhang, Yang Jiao, et al. Evaluation of prevention and control measures for a waterborne norovirus outbreak in Beijing based on dynamic model. Dis Surveill, 2023, 38(8):1007–1013. doi: 10.3784/jbjc.20230 .5150220

[12] Rong Hu. Epidemic characteristics of an outbreak of human adenovirus infection in a kindergarten based on infectious disease dynamics model. Pract Prev Med,2023,30(9):1087–1090. doi: 10.3969/j.Issn.1006-3110.2023.09.014

[13] The Joint Prevention and Control Mechanism of The State Council for the novel coronavirus pneumonia epidemic. Novel Coronavirus Pneumonia Prevention and Control Protocol (ninth edition).https://www.gov.cn/xinwen/2022-06/28/content_5698168.htm

[14] juan-juan Zhang, qian-hui Wu, hong-jie Yu. Research Progress of Epidemiology, transmission dynamics, Vaccine and non-drug intervention evaluation of COVID-19. Bulletin of National natural science foundation of China, 2022, 36(4):122–133. doi: 10.16262/j.cnki.1000-8217.20220829.008

[15] WHO. Weekly epidemiological update on COVID-19–25 May 2023. https://www.who.int/publications/m/item/weekly-epidemiological-update-on-covid-19—25-may-2023.

[16] Min Zhang, Wen-juan Yan. Spatial and temporal scanning analysis of the epidemic situation of COVID-19 in Pudong New Area, Shanghai from January to June 2022. Modern Preventive Medicine, 2023, 50 (5):786–791.

[17] Fang Shi, Hao-yu Wen, Rui Liu, Jian-jun Bai, Fang Wang, Mubarik Sumaira, et al. The comparison of epidemiological characteristics between confirmed and clinically diagnosed cases with COVID-19 during the early epidemic in Wuhan, China. Glob Health Res Policy, 2021 May 28;6(1):18. doi: 10.1186/s41256-021-00200-8 34049599 PMC8161348

[18] Ji-chao Sha, Cui-da Meng, Jing Sun, Li-wei Sun, Rui Gu, Jun-zhi Liu, et al. Clinical and upper airway characteristics of 3715 patients with the Omicron variant of SARS-Cov-2 in Changchun, China. J Infect Public Health,2023, 16(3):422–429. Epub 2023 Jan 20. doi: 10.1016/j.jiph.2023.01.013 36731245 PMC9854235

[19] Alsayed Abdallah, Sadir Hayder, Kamil Raja, Sari Hasan. Prediction of Epidemic Peak and Infected Cases for COVID-19 Disease in Malaysia, 2020. Int. J. Environ. Res. Public Health 2020, 17, 4076; doi: 10.3390/ijerph17114076 32521641 PMC7312594

[20] Bartolomeo Nicola, Trerotoli Paolo, Serio Gabriella. Short-term forecast in the early stage of the COVID-19 outbreak in Italy. Application of a weighted and cumulative average daily growth rate to an exponential decay model. Infectious Disease Modelling, 2021 (6) 212–221. 33398249 10.1016/j.idm.2020.12.007 PMC7773318

[21] Mangla Sherry, Pathak Ashok Kumar, Arshad Mohd. and Haque Ubydul. Short-term forecasting of the COVID-19 outbreak in India. International Health 2021; 13: 410–420. doi: 10.1093/inthealth/ihab031 34091670 PMC8194983

[22] Wu Ze-yang, Hong Bo-zhang, Hong Fei-zhao. Modeling of the Small-Scale Outbreak of COVID-19. Front. Public Health 10:907814. doi: 10.3389/fpubh.2022.907814 35844852 PMC9283974

[23] Wei-zhong Yang, Ting Zhang. Strategy and measures in response to highly uncertain emerging infectious disease. Chin J Epidemiol, 2022, 43 (5):627–633. 10.3760/cma.j.cn112338-20220210-00106 35589564

[24] Qin Li, Fei Kong, Yuan-yuan Wang. Screening and Establishment of Indicators for Suspected Early Warning Features of Emerging Infectious Diseases. Chinese Hospital Management, 2022, 42(3): 6–8.

[25] Li-li Jiang. The application of high-throughput sequencing technology for pathogen detection in emerging infectious diseases. Labeled immunoassays & ClinMed, 2022, 29(5):897–900.

